# Small Vulnerable Newborns Among Venezuelan Immigrants in Colombia Between 2018 and 2022

**DOI:** 10.1007/s10903-025-01842-3

**Published:** 2026-01-16

**Authors:** Paula Andrea Castro-Prieto, Paola Rueda-Guevara, Maida Juni

**Affiliations:** 1 Urban Health Collaborative, Drexel Dornsife School of Public Health, Drexel University, Philadelphia, USA; 2 Population Studies Center and Department of Sociology, University of Pennsylvania, Philadelphia, USA; 3 International Max Planck Research School for Population, Max Planck Institute for Demographic Research, Rostock, Germany; 4 Centre d’Estudis Demogràfics, Autonomous University of Barcelona, Cerdanyola del Vallès, Spain

**Keywords:** Small vulnerable newborns, Low birth weight, Preterm birth, Human migration

## Abstract

Small Vulnerable Newborns (SVNs)—including low birth weight (LBW), preterm birth (PB), and small for gestational age (SGA)—represent a major global concern, with 35.3 million cases reported in 2020. Despite the growing overlap between migration and perinatal health, evidence on birth outcomes among Venezuelan migrants in Colombia remains limited. This study examined the association between maternal nationality and the likelihood of SVN, LBW, and PB among live births in Colombia from 2018 to 2022. We conducted a cross-sectional analysis of 3,111,716 births using national vital statistics (DANE). The primary exposure was maternal nationality. Outcomes included LBW (< 2,500 g), PB (< 37 weeks), and the combined SVN indicator. Logistic regression models were adjusted for sociodemographic variables (maternal age, education, marital status, region, newborn sex, and year), followed by additional adjustment for maternal factors (parity, interbirth interval, multiple births). Interaction models were also developed between maternal origin and maternal education, marital status, region, and interbirth interval. After sociodemographic and maternal-factor adjustment, Venezuelan mothers had higher odds of SVN (OR: 1.18, 95% CI: 1.13–1.22), LBW (OR: 1.19, 95% CI: 1.15–1.24), and PB (OR 1.06, 95% CI: 1.02–1.10). Interaction analyses showed that associations varied across geographic, educational, and reproductive contexts. For SVN, differences emerged particularly among women with missing education, missing marital status, and shorter interbirth intervals. These findings highlight the vulnerability of migrant mothers and underscore the need for equity-oriented maternal and child health policies in host countries.

**Supplementary Information:**

The online version contains supplementary material available at 10.1007/s10903-025-01842-3.

## Introduction

Small Vulnerable Newborns (SVN) represent a significant infant and maternal health concern, encompassing Preterm Birth (PB), Small for Gestational Age (SGA), or with Low Birth Weight (LBW). In 2020, out of 135 million live births, 35.3 million were classified as SVN worldwide [1, 2]. Despite underlying mechanisms and clinical management differences, PB, SGA, and LBW share common risk factors, causes, and consequences. Each condition reflects some form of restricted growth, whether in gestational duration as PB, absolute size as LBW, or size relative to gestational age as SGA [3]. Adverse outcomes can manifest in all three conditions that make up SVN or at least in one. During childhood, these conditions are associated with an increased risk of mortality, stunting, wasting, cerebral palsy, epilepsy, reduced brain volume, and lower IQ and cognitive performance, among other conditions. In adulthood, they may increase the risk of developing depression, anxiety, metabolic syndrome, diabetes, hypertension, coronary disease, stroke, and other health issues [1].

Evidence highlights contextual factors that are related to the increase in maternal and fetal vulnerability to SVN. These factors include political instability and armed conflict [1, 4], inadequate water sanitation and hygiene, environmental degradation, limited access to healthcare, low educational attainment, and sociocultural influences. These determinants contribute to risk, including undernutrition, infections, extreme maternal age (both early and advanced), extreme parity, and short pregnancy intervals [1, 5, 6]. Migration represents an important contextual determinant that can influence perinatal outcomes for mothers and their newborns. Although vulnerable SVNs share common causes and consequences, migration adds a dimension of vulnerability.

Migration is a significant contextual factor influencing birth outcomes, though its effects may vary depending on generational status, length of residence, and country of settlement [7–9]. For instance, studies in the United States showed that second-generation Latinas and long-term residents tend to experience poorer birth outcomes, a pattern also observed among Puerto Rican and Black immigrant populations. Similarly, among Mexican-origin women, those born and giving birth in Mexico have a 37% to 64% lower risk of PB or LBW compared to U.S.-born Mexican Americans [7]. In contrast, some European countries report a “healthy migrant effect”, where certain immigrant groups exhibit better birth outcomes than the native-born population. For example, Somali migrants in Belgium, Canada, Finland, Norway, and Sweden have demonstrated more favorable birth outcomes [8]. Further highlighting the role of migration policies, a comparative study found that Latin American immigrants in Canada had better birth outcomes than those in Spain, suggesting that selective migration may influence maternal and neonatal health [9]. These international and generational patterns suggest that migration alone does not fully explain differences in birth outcomes. Instead, they highlight the importance of understanding how the complex interplay between migration, social conditions in the host country, and broader socioeconomic and environmental factors can influence maternal and infant health outcomes.

In recent years, Latin America has experienced a considerable wave of migration, with Venezuela as the main originator of the migrant population. Millions have fled the country due to political and economic issues, insecurity, widespread hunger, high stress levels, uncertainty, and lack of access to essential medicines and medical care [10–12]. According to the Coordination for Venezuelan Refugees and Immigrants and the International Organization for Migration, at least 7.8 million Venezuelans had emigrated by 2024, with Colombia hosting approximately 2.8 million—51% are women [13, 14]. This migration wave has been characterized by a growing feminization of migration. Between 2018 and 2019, Colombia’s Migration Office in Norte de Santander recorded data from approximately 12,000 women awaiting regularized entry, 40% of whom reported having a bachelor’s degree. The primary reasons for leaving Venezuela included insufficient income to meet basic needs, violence, hunger, health challenges, and the search for better job opportunities [15]. These circumstances pose significant challenges to birth outcomes, particularly in Colombia, a country unaccustomed to large-scale immigration.

Despite the critical intersection of perinatal outcomes and migration, research on perinatal outcomes among Venezuelan women in Colombia remains limited. To date, only one study has examined this association in the country. A 2017 study reported that Venezuelan mothers had higher rates of LBW, and lower Apgar scores compared to Colombian mothers [16]. However, significant knowledge gaps persist regarding outcomes from 2018 to 2022. Therefore, we aim to analyze the association between SVN—with a focus on LBW and PB—and maternal origin among Colombian newborns between 2018 and 2022.

## Methods

### Data Collection

This cross-sectional study was based on data from the Vital Statistics compiled by the National Administrative Department of Statistics in Colombia (DANE, by its Spanish acronym). DANE compiles the national births registry, which contains data on live births recorded annually and reported by health personnel based on mandatory individual records completed at the institutions where births occur. The information is publicly accessible [17]. We used data from January 1, 2018, to January 1, 2023, covering all live births from 2018 to 2022. The database contained 3,111,716 live births during the study period, distributed as follows: 649,115 in 2018, 642,660 in 2019, 629,402 in 2020, 616,914 in 2021, and 573,625 in 2022.

### Variables Definition

The primary *exposure* was maternal origin, derived from the original variable “country of habitual residence reported for the mother.” This variable was used as a proxy of maternal nationality, as it is the only one that captures information on the mothers’ country of origin and potential migration status, particularly relevant during this period of substantial migration flows from Venezuela to Colombia. For analysis purposes, births from mothers whose habitual residence was outside of Colombia or Venezuela were excluded. Outcomes variables were: LBW, PB, and the combined occurrence of both LBW and BP (SVN). LBW is defined as a weight of less than 2,500 g, and PB is defined as a delivery before 37 gestational weeks. Sociodemographic covariates included from the dataset were: maternal age groups (10–19, 20–34, ≥ 35), maternal education (primary, secondary, technical and technological, professional level and higher, no information), marital status (married or cohabiting, single/divorced/widow, no information), region of residence (Bogota, Caribe, Central, Oriental, Pacifica, Orinoquia/Amazonia), newborn sex (female, male) and year of birth (2018, 2019, 2020, 2021, 2022). Maternal factors were number of previous pregnancies (1, 2–4, ≥5), interbirth interval (primiparous, 0–2 years, 2.1-5 years, > five years), multiple births (yes, no). Covariates were selected based on previous literature identifying them as factors associated with the outcomes [1, 2, 16].

It should be noted that the DANE Vital Statistics system does not collect detailed clinical variables, including information on maternal comorbidities, nutritional status, substance use, or obstetric complications. Consequently, these factors could not be incorporated into the analytical models.

### Data Analysis

The analysis was conducted in three sequential stages. First, descriptive statistics were computed for all variables to provide an overview of the sample. Second, chi-squared tests were applied to assess significant differences in variables by nationality. Third, multivariable logistic regression models were estimated in three steps, progressively adjusting for additional covariates to evaluate the independent effect of birth outcomes while controlling for potential confounders. Model 1 included maternal origin, Model 2 incorporated sociodemographic variables, and Model 3 added maternal factors. These models were performed separately for each birth outcome (SVN, LBW, and PB). Missing data was minimal (0.3%) and managed through case-wise deletion. Covariates were selected based on prior literature, and sensitivity analyses were performed to assess the stability of estimates across models. Given that effects may vary across subgroups, interaction terms were included between maternal origin and key predictors such as region, education, marital status, previous pregnancies, and interpregnancy interval. Logistic regression was used given its suitability for binary outcomes and its ability to estimate adjusted odds ratios in large population datasets. Associations were reported as odds ratios (ORs) with 95% confidence intervals (CIs), and all analyses were conducted in R.

### Ethical Approval

This study adheres to the principles of the Declaration of Helsinki on research involving human participants. It consisted solely of a secondary analysis of anonymized, publicly available data published by the DANE and therefore posed minimal risk. In Colombia, research using anonymized public datasets without any personal identifiers is typically considered exempt from ethical review under Resolution 8430 of 1993.

## Results

The final dataset included 3,102,471 records, distributed as follows: 648,005 in 2018, 640,415 in 2019; 626,603 in 2020; 616,224 in 2021; and 571,224 in 2022. A total of 0.3% points were excluded from the final sample due to missing data in key covariates, including maternal age, education, and marital status.

Colombian and Venezuelan mothers were mostly aged 20–34 years (70.05% vs. 66.35%). Regarding education, secondary schooling was more common among Venezuelan mothers (63.96% vs. 58.52%), whereas professional or higher education was more frequent among Colombians (12.26% vs. 3.87%). Additionally, Venezuelan mothers more often had over five previous pregnancies (9.89% vs. 5.47%) and shorter interbirth intervals (14.40% vs. 7.14%). Regionally, although most births occurred in the Caribbean, the Oriental region accounted for a substantially larger share among Venezuelan mothers (34.77% vs. 16.02%). In terms of prevalence, the selected outcomes—SNV (9.13% vs. 8.95%), LBW (9.54% vs. 9.41%), and PB (10.05% vs. 9.92%)—were slightly higher among Colombian mothers compared with Venezuelan mothers (Table 1). Year-by-year results showed that in 2018 and 2019 the outcomes under study had higher prevalences among children of Venezuelan mothers; however, from 2020 onward, prevalences were slightly higher among children of Colombian mothers. Detailed year-stratified descriptive results are provided in Appendix 1.

**Table 1:** Sociodemographic and birth outcomes of National Births in Colombia between 2018 and 2022

Variable	Colombia		Venezuela		*p*-value
	*n*	%	*n*	%	
*Maternal age groups (years)*					
10–19	566,224	18.48	10,478	27.68	< 0.0001
20–34	2,146,794	70.05	25,122	66.35	
≥ 35	351,593	11.47	2260	5.97	
*Maternal education*					
Primary or less	397,305	12.96	8059	21.29	< 0.0001
Secondary	1,793,297	58.52	24,215	63.96	
Technical and technological	390,558	12.74	805	2.13	
Professional or higher	375,576	12.26	1464	3.87	
No information	107,875	3.52	3317	8.76	
*Marital status*					
Married or cohabitation	2,590,468	84.53	29,499	77.92	< 0.0001
Single/divorced/widow	394,886	12.89	5120	13.52	
No information	79,257	2.59	3241	8.56	
*Region*					
Bogota D.C	454,497	14.83	1061	2.8	< 0.0001
Caribe	922,802	30.11	13,870	36.63	
Central	671,369	21.91	336	0.89	
Oriental	491,034	16.02	13,163	34.77	
Pacifica	423,816	13.83	113	0.3	
Orinoquia, Amazonia	101,093	3.3	9317	24.61	
*Newborn sex*					
Male	1,570,492	51.25	19,662	51.93	0.0079
Female	1,494,119	48.75	18,198	48.07	
*Number of previous pregnancies*					
1	1,250,753	40.81	13,102	34.61	< 0.0001
2–4	1,646,087	53.71	21,015	55.51	
≥5	167,771	5.47	3743	9.89	
*Interbirth interval*					
0–2 years	218,855	7.14	5453	14.4	< 0.0001
2.1 years − 5 years	649,661	21.2	12,317	32.53	
> 5 years	798,079	26.04	5624	14.85	
Primiparous	1,398,016	45.62	14,466	38.21	
*Multiple birth*					
Yes	53,593	1.75	755	1.99	0.0003
No	3,011,018	98.25	37,105	98.01	
*SVN*					
Yes	2,784,734	90.87	34,472	91.05	0.2206
No	279,877	9.13	3388	8.95	
*LBW*					
Yes	2,772,397	90.46	34,297	90.59	0.4188
No	292,214	9.54	3563	9.41	
*PB*					
Yes	2,756,504	89.95	34,106	90.08	0.3786
No	308,107	10.05	3754	9.92	
*Survey year*					
2018	644,147	21.02	3858	10.19	< 0.0001
2019	628,990	20.52	11,425	30.18	
2020	616,930	20.13	9673	25.55	
2021	607,513	19.82	8711	23.01	
2022	567,031	18.5	4193	11.08	

Regarding the logistic regression models, in Model 1, adjusting for maternal nationality, no statistically significant differences were found between Colombian and Venezuelan mothers across the three outcomes. However, in Model 2, which adjusted for sociodemographic characteristics, including maternal age, education level, marital status, region of residence, newborn sex, and year of birth, Venezuelan mothers were 19% more likely to experience SVNs outcomes, 20% more likely to have a newborn with LBW, and 10% more likely to experience PB. Finally, in Model 3, which further adjusted for maternal factors such as previous pregnancies, interbirth interval, and multiple births, Venezuelan mothers remained at increased risk, 18% more likely to experience SVNs, 19% for LBW, and 6% for PB (See Table 2).

**Table 2:** Association between maternal origin and Small Vulnerable Newborns, Low Birth Weight, Preterm Birth among Colombian Newborns, 2018–2022

	Model 1^a^ (adjusted for maternal origin)	Model 2^b^ (model 1 + sociodemographic variables)	Model 3^c^ (model 2 + maternal factors)
*Small Vulnerable Newborns (SVN)*			
Maternal Origin			
Colombia	Referent	Referent	Referent
Venezuela	0.98 (0.94–1.01)	1.19 (1.14–1.23)***	1.18 (1.13–1.22)***
*Low Birth Weight (LBW)*			
Maternal Origin			
Colombia	Referent	Referent	Referent
Venezuela	0.99 (0.95–1.02)	1.20 (1.16–1.25)***	1.19 (1.15–1.24)***
*Preterm Birth (PB)*			
Maternal Origin			
Colombia	Referent	Referent	Referent
Venezuela	0.98 (0.95–1.02)	1.10 (1.07–1.14)***	1.06 (1.02–1.10)**

*p*-value: ***≤0.001, **≤0.01, *≤0.05

^a^Model 1: adjusted for maternal origin (Colombia, Venezuela)

^b^Model 2: Model 1 plus maternal age groups, maternal education, marital status, region of residence, newborn sex, and year of birth

^c^Model 3: Model 2 plus number of previous pregnancies, interbirth interval, and multiple births

When interaction terms were introduced, the association between nationality and the outcomes weakened or became non-significant in several models, showing variation across subgroups. For SVN, significant interactions were found with region, maternal education, marital status, and interbirth interval, with stronger nationality-related differences in Orinoquía/Amazonía—the least populated region of the country—and among women with professional or missing education or missing marital status. Nationality was no longer significant in the regional model but remained significant in the education and marital status models. Similar patterns were observed for BPN, nationality remained significant in the education and missing–marital-status interactions, and short interbirth interval (0–2 years) also showed significant interactions. In contrast, nationality was not significant in the regional model, despite persistent differences in Orinoquía and Amazonía. For PB, nationality was not significant overall, although interactions appeared for region, education, missing marital status, and short interbirth interval.

Overall, these findings show that the association between nationality and adverse birth outcomes differs across geographic, educational, and reproductive contexts (See Appendix 2).

## Discussion

This study examined perinatal outcomes among Colombian and Venezuelan mothers between 2018 and 2022, focusing on SVN, LBW, and PB. Venezuelan mothers showed higher odds of all three outcomes, even after adjustment. After controlling sociodemographic factors, they were 19% more likely to experience SVN, 20% more likely to have LBW, and 10% more likely to have PB; these estimates decreased slightly after adjusting for maternal factors. Although the overall models indicate elevated risks among Venezuelan mothers, interaction analyses revealed that these associations vary across subgroups. Differences by region, education, marital status, and reproductive history highlight the role of contextual vulnerabilities shaping perinatal outcomes.

As expected, after adjusting for sociodemographic variables, such as maternal age, educational attainment, and marital status, the odds of experiencing SVNs, LBW, and PB increased. Among these variables, maternal age is a well-established factor influencing perinatal health. Younger mothers are at increased risk of obstetric complications such as preeclampsia, fetal growth restriction, and PB [18], while older mothers may experience a decline in oocyte quality, which can negatively affect fertility and pregnancy outcomes [19]. Educational attainment also plays a protective role. In the Colombian context, higher levels of educational attainment have been associated with a lower risk of adverse perinatal outcomes [20]. However, the interaction analyses showed that Venezuelan mothers with the highest educational levels, as well as those with missing educational information, had particularly elevated odds of the selected outcomes. Although education often serves as a proxy for socioeconomic vulnerability, which is known to directly impact newborn health, it is important to acknowledge that the migration flow from Venezuela to Colombia has produced a heterogeneous educational profile. As a result, Venezuelan mothers do not necessarily follow the expected pattern of uniformly low educational attainment. Likewise, marital status emerged as a relevant factor. Although the study did not include detailed information on household structure (e.g., nuclear vs. extended families), the findings suggest that being single, divorced, or widowed may increase maternal vulnerability. This is particularly relevant for single-parent households, especially those headed by women, which are at greater risk of poverty compared to those with two caregivers [21].

In addition to sociodemographic characteristics, maternal health-related factors were also associated with increased odds of SVNs, LBW, and PB. Specifically, shorter interbirth intervals, primiparous, high parity, and multiple births were linked to poorer perinatal outcomes, consistent with prior evidence [22–24].

Beyond maternal factors, migration plays a key role in shaping perinatal health. This vulnerability increases when economic constraints and systemic barriers limit access to maternal healthcare [25]. In our study, children of Venezuelan mothers had higher odds of SVN, LBW, and PB, even after adjusting for sociodemographic and obstetric factors. Evidence from other settings is mixed. In contrast, studies from Australia and the United States report higher rates of LBW and poorer birth outcomes among certain migrant groups, including Indian-born women [26], second-generation Latinas, Puerto Rican, and Black immigrants [7]. European findings also vary in Belgium, migrant women exhibited higher perinatal mortality linked to socioeconomic disadvantage, yet migration appeared protective against LBW among low-SES women [27]. Over time, LBW rates increased among Maghrebi mothers, although they remained lower even after a decade of Belgian nationality [28]. Additionally, undocumented women faced higher perinatal risks, with causes varying by nationality [29].

Some studies report more favorable outcomes among migrants. In Canada, uninsured pregnant migrants received inadequate prenatal care yet showed no differences in LBW or PB [30]. In Brazil, migrant mothers generally had better perinatal outcomes, though Indigenous and Black women continued to face barriers to antenatal care [31].

In Colombia, Garnica-Rosas et al. (2021) found that Venezuelan mothers had higher odds of LBW compared to Colombian mothers [16]. However, their analysis included data from 2017, while the highest peaks of Venezuelan immigration were recorded after 2018 [32]. Our study, covering data from 2018 to 2022, suggests that the increased odds of adverse perinatal outcomes among Venezuelan women may be attributed to contextual factors. Many migrated due to insufficient income, violence, hunger, the search for better job opportunities, and health challenges [15]. In this context, access to healthcare emerges as a critical issue, particularly regarding prenatal and postnatal care for Venezuelan women [33]. Additionally, reaching Colombia also entails significant risks. A qualitative study on the Colombia-Venezuela border reported exposure to armed-group control, lack of state protection, and vulnerabilities that foster exploitation, including transactional sex. Limited social resources and restricted access to healthcare further increase migrants’ vulnerability in the host country. These risks intensified during COVID-19 border closures [34]. Although all three outcomes studied showed lower levels among children of Venezuelan mothers than among those of Colombian mothers from 2020 onward.

Our results suggest that Venezuelan mothers had higher odds of having newborns with SVNs, LBW, and PB compared to Colombian mothers. These disparities may be partially explained by the social conditions to which Venezuelan mothers were previously exposed in their country of origin, as well as by the initial response strategies implemented by the Colombian government during the early stages of the migration wave. Colombia’s policy response also played a role in shaping health access for immigrants. In 2019, the government implemented measures such as childbirth coverage through public resources and laws granting Colombian nationality to newborns, ensuring access to essential services such as healthcare, social support, and education [35, 36]. Currently, 2.351.663 Venezuelans reside in Colombia with regular immigration status, meaning that they hold a valid authorization of stay in the country [13]. However, healthcare challenges existed even before the immigration wave. The Comprehensive Maternal Perinatal Health Care Route (Rutas de Atencion Materno Perinatal in Spanish), established in 2018 [37], has not fully guaranteed comprehensive care for mothers and newborns. Evidence has highlighted several barriers, including low adherence among healthcare professionals due to a lack of updated training, limited access to family planning and appointment scheduling, and geographical constraints [38–40]. It is important to note that our results indicate an increase in the odds of SVN, LBW, and PB between 2018 and 2022, highlighting the need to reconsider these measures and integrate migration into intervention strategies. Identifying these factors may help inform policymakers in developing targeted strategies to improve perinatal outcomes, particularly in the context of ongoing migration flows.

We acknowledge several limitations. First, the study examines Colombia’s first major migration wave, a period marked by substantial governmental and social challenges that were not fully captured in available data. Second, the migration crisis intensified during the pandemic, further complicating the context for migrant mothers. Third, key migration-related variables—such as duration of residence in Colombia, migration status, and acculturation—were not included because they are not collected in the birth registry [41]. Finally, the cross-sectional nature of the data prevents establishing temporality or causal relationships. Future research should incorporate these dimensions to deepen the understanding of SVN among immigrant populations.

The main contribution of this study lies in its ability to examine the association between maternal nationality and SVNs, focusing on LBW and PB, among Colombian newborns during a critical period of migration to Colombia, an aspect not previously explored in this way. This understanding is essential for developing targeted public health strategies aimed at populations affected by SVN conditions, including maternal and infant groups. However, addressing perinatal health in migrant populations requires moving beyond individual-level factors to consider the broader social context. This includes recognizing how poverty, socioeconomic status, and education influence health outcomes, as well as understanding migrants’ needs, cultural beliefs, and health-seeking behaviors within host countries. Such a comprehensive perspective is crucial for developing effective, culturally sensitive policies and programs that can help mitigate long-term health, social, and economic consequences. Finally, the applicability of these findings should be considered within the Colombian context, as differences in health systems, migration policies, and data quality across countries may influence the observed associations. These results are therefore most relevant for Latin American settings with similar conditions.

## Supplementary Information

Below is the link to the electronic supplementary material.

Supplementary Material 1

Supplementary Material 2

## Data Availability

The data used in this study are openly accessible and correspond to Colombia’s vital statistics for the years 2018–2022. These data are collected and curated by the National Administrative Department of Statistics (DANE, by its Spanish acronym) and are available for public download through the official DANE website.
