# Supplementary material for: Small Vulnerable Newborns Among Venezuelan Immigrants in Colombia Between 2018 and 2022
Source: J Immigr Minor Health. 2026 Jan 16;28(4):916–23. doi: 10.1007/s10903-025-01842-3 (PMC13451447; doi:10.1007/s10903-025-01842-3)
Supplement: Supplementary file 1 [file 10903_2025_1842_MOESM1_ESM.docx]

**Appendix 1. Sociodemographic and birth outcomes of National Births in Colombia between 2018 and 2022**

|  | **2018** | | | | | **2019** | | | | | **2020** | | | | | **2021** | | | | | **2022** | | | | |
| --- | --- | --- | --- | --- | --- | --- | --- | --- | --- | --- | --- | --- | --- | --- | --- | --- | --- | --- | --- | --- | --- | --- | --- | --- | --- |
|  | **Colombia** | | **Venezuela** | | **p-value** | **Colombia** | | **Venezuela** | | **p-value** | **Colombia** | | **Venezuela** | | **p-value** | **Colombia** | | **Venezuela** | | **p-value** | **Colombia** | | **Venezuela** | | **p-value** |
| **Variable** | **n** | **%** | **n** | **%** |  | **n** | **%** | **n** | **%** |  | **n** | **%** | **n** | **%** |  | **n** | **%** | **n** | **%** |  | **n** | **%** | **n** | **%** |  |
| **Maternal age groups (years)** | |  |  |  |  |  |  |  |  |  |  |  |  |  |  |  |  |  |  |  |  |  |  |  |  |
| 10-19 | 127689 | 19.82 | 905 | 23.46 | <0.0001 | 119818 | 19.05 | 3055 | 26.7 | <0.0001 | 112156 | 18.18 | 2729 | 28.21 | <0.0001 | 110021 | 18.11 | 2545 | 29.22 | <0.0001 | 96540 | 17.03 | 1244 | 29.67 | <0.0001 |
| 20-34 | 444764 | 69.05 | 2678 | 69.41 |  | 438387 | 69.7 | 7721 | 67.6 |  | 434647 | 70.45 | 6403 | 66.19 |  | 427101 | 70.3 | 5661 | 64.99 |  | 401895 | 70.88 | 2659 | 63.42 |  |
| >35 | 71694 | 11.13 | 275 | 7.13 |  | 70785 | 11.25 | 649 | 5.68 |  | 70127 | 11.37 | 541 | 5.59 |  | 70391 | 11.59 | 505 | 5.8 |  | 68596 | 12.1 | 290 | 6.92 |  |
| **Maternal education** |  |  |  |  |  |  |  |  |  |  |  |  |  |  |  |  |  |  |  |  |  |  |  |  |  |
| Primary or less | 86439 | 13.42 | 782 | 20.27 | <0.0001 | 81504 | 12.96 | 1994 | 17.5 | <0.0001 | 78481 | 12.72 | 2170 | 22.43 | <0.0001 | 81080 | 13.35 | 2081 | 23.89 | <0.0001 | 69801 | 12.31 | 1032 | 24.61 | <0.0001 |
| Secondary | 373532 | 57.99 | 2260 | 58.58 |  | 366836 | 58.32 | 6638 | 58.1 |  | 364391 | 59.07 | 6680 | 69.06 |  | 359100 | 59.11 | 5919 | 67.95 |  | 329438 | 58.1 | 2718 | 64.82 |  |
| Technical and technological | 80489 | 12.5 | 132 | 3.42 |  | 80250 | 12.76 | 295 | 2.58 |  | 77069 | 12.49 | 196 | 2.03 |  | 75291 | 12.39 | 134 | 1.54 |  | 77459 | 13.66 | 48 | 1.14 |  |
| Professional or higher | 77397 | 12.02 | 216 | 5.6 |  | 77336 | 12.3 | 455 | 3.98 |  | 74195 | 12.03 | 349 | 3.61 |  | 72072 | 11.86 | 297 | 3.41 |  | 74576 | 13.15 | 147 | 3.51 |  |
| No information | 26290 | 4.08 | 468 | 12.13 |  | 23064 | 3.67 | 2043 | 17.9 |  | 22794 | 3.69 | 278 | 2.87 |  | 19970 | 3.29 | 280 | 3.21 |  | 15757 | 2.78 | 248 | 5.91 |  |
| **Marital status** |  |  |  |  |  |  |  |  |  |  |  |  |  |  |  |  |  |  |  |  |  |  |  |  |  |
| Married or cohabitation | 537122 | 83.39 | 2818 | 73.04 | <0.0001 | 528288 | 83.99 | 7568 | 66.2 | <0.0001 | 521097 | 84.47 | 8083 | 83.56 | 0.0344 | 517548 | 85.19 | 7471 | 85.77 | <0.0001 | 486413 | 85.78 | 3559 | 84.88 | 0.0019 |
| Single/divorced/widow | 88506 | 13.74 | 610 | 15.81 |  | 83820 | 13.33 | 1616 | 14.1 |  | 78925 | 12.79 | 1296 | 13.4 |  | 74378 | 12.24 | 1080 | 12.4 |  | 69257 | 12.21 | 518 | 12.35 |  |
| No information | 18519 | 2.87 | 430 | 11.15 |  | 16882 | 2.68 | 2241 | 19.6 |  | 16908 | 2.74 | 294 | 3.04 |  | 15587 | 2.57 | 160 | 1.84 |  | 11361 | 2 | 116 | 2.77 |  |
| **Region** |  |  |  |  |  |  |  |  |  |  |  |  |  |  |  |  |  |  |  |  |  |  |  |  |  |
| Bogota D.C | 103110 | 16.01 | 429 | 11.12 | <0.0001 | 100455 | 15.97 | 618 | 5.41 | <0.0001 | 92755 | 15.03 | 9 | 0.09 | <0.0001 | 79795 | 13.13 | 2 | 0.02 | <0.0001 | 78382 | 13.82 | 3 | 0.07 | <0.0001 |
| Caribe | 187736 | 29.14 | 1561 | 40.46 |  | 187608 | 29.83 | 4456 | 39 |  | 183040 | 29.67 | 3552 | 36.72 |  | 189702 | 31.23 | 2980 | 34.21 |  | 174716 | 30.81 | 1321 | 31.5 |  |
| Central | 141760 | 22.01 | 71 | 1.84 |  | 136602 | 21.72 | 196 | 1.72 |  | 134418 | 21.79 | 45 | 0.47 |  | 133157 | 21.92 | 6 | 0.07 |  | 125432 | 22.12 | 18 | 0.43 |  |
| Oriental | 101362 | 15.74 | 1251 | 32.43 |  | 100513 | 15.98 | 3599 | 31.5 |  | 100066 | 16.22 | 3643 | 37.66 |  | 96821 | 15.94 | 3209 | 36.84 |  | 92272 | 16.27 | 1461 | 34.84 |  |
| Pacifica | 90089 | 13.99 | 11 | 0.29 |  | 84458 | 13.43 | 58 | 0.51 |  | 86655 | 14.05 | 18 | 0.19 |  | 86556 | 14.25 | 11 | 0.13 |  | 76058 | 13.41 | 15 | 0.36 |  |
| Orinoquia, Amazonia | 20090 | 3.12 | 535 | 13.87 |  | 19354 | 3.08 | 2498 | 21.9 |  | 19996 | 3.24 | 2406 | 24.87 |  | 21482 | 3.54 | 2503 | 28.73 |  | 20171 | 3.56 | 1375 | 32.79 |  |
| **Newborn sex** |  |  |  |  |  |  |  |  |  |  |  |  |  |  |  |  |  |  |  |  |  |  |  |  |  |
| Male | 330178 | 51.26 | 2011 | 52.13 | 0.2899 | 322910 | 51.34 | 5964 | 52.2 | 0.0687 | 316457 | 51.3 | 5029 | 51.99 | 0.1783 | 311139 | 51.22 | 4530 | 52 | 0.147 | 289808 | 51.11 | 2128 | 50.75 | 0.6548 |
| Female | 313969 | 48.74 | 1847 | 47.87 |  | 306080 | 48.66 | 5461 | 47.8 |  | 300473 | 48.7 | 4644 | 48.01 |  | 296374 | 48.78 | 4181 | 48 |  | 277223 | 48.89 | 2065 | 49.25 |  |
| **Number of previous pregnancies** | | |  |  |  |  |  |  |  |  |  |  |  |  |  |  |  |  |  |  |  |  |  |  |  |
| 1 | 275053 | 42.7 | 1296 | 33.59 | <0.0001 | 259881 | 41.32 | 3941 | 34.5 | <0.0001 | 250897 | 40.67 | 3359 | 34.73 | <0.0001 | 238936 | 39.33 | 3019 | 34.66 | <0.0001 | 225986 | 39.85 | 1487 | 35.46 | <0.0001 |
| 2-4 | 334681 | 51.96 | 2177 | 56.43 |  | 335131 | 53.28 | 6423 | 56.2 |  | 333101 | 53.99 | 5402 | 55.85 |  | 333430 | 54.88 | 4751 | 54.54 |  | 309744 | 54.63 | 2262 | 53.95 |  |
| >=5 | 34413 | 5.34 | 385 | 9.98 |  | 33978 | 5.4 | 1061 | 9.29 |  | 32932 | 5.34 | 912 | 9.43 |  | 35147 | 5.79 | 941 | 10.8 |  | 31301 | 5.52 | 444 | 10.59 |  |
| **Interbirth interval** |  |  |  |  |  |  |  |  |  |  |  |  |  |  |  |  |  |  |  |  |  |  |  |  |  |
| 0-2 years | 45273 | 7.03 | 536 | 13.89 | <0.0001 | 45299 | 7.2 | 1626 | 14.2 | <0.0001 | 43597 | 7.07 | 1365 | 14.11 | <0.0001 | 46558 | 7.66 | 1324 | 15.2 | <0.0001 | 38128 | 6.72 | 602 | 14.36 | <0.0001 |
| 2.1 years - 5 years | 134185 | 20.83 | 1269 | 32.89 |  | 135776 | 21.59 | 3915 | 34.3 |  | 133525 | 21.64 | 3163 | 32.7 |  | 131326 | 21.62 | 2707 | 31.08 |  | 114849 | 20.25 | 1263 | 30.12 |  |
| >5 years | 159378 | 24.74 | 609 | 15.79 |  | 158942 | 25.27 | 1578 | 13.8 |  | 159959 | 25.93 | 1402 | 14.49 |  | 161254 | 26.54 | 1334 | 15.31 |  | 158546 | 27.96 | 701 | 16.72 |  |
| Primiparous | 305311 | 47.4 | 1444 | 37.43 |  | 288973 | 45.94 | 4306 | 37.7 |  | 279849 | 45.36 | 3743 | 38.7 |  | 268375 | 44.18 | 3346 | 38.41 |  | 255508 | 45.06 | 1627 | 38.8 |  |
| **Multiple birth** |  |  |  |  |  |  |  |  |  |  |  |  |  |  |  |  |  |  |  |  |  |  |  |  |  |
| Yes | 11283 | 1.75 | 73 | 1.89 | 0.5473 | 10729 | 1.71 | 217 | 1.9 | 0.1222 | 10732 | 1.74 | 190 | 1.96 | 0.1018 | 10527 | 1.73 | 175 | 2.01 | 0.0552 | 10322 | 1.82 | 100 | 2.38 | 0.0077 |
| No | 632864 | 98.25 | 3785 | 98.11 |  | 618261 | 98.29 | 11208 | 98.1 |  | 606198 | 98.26 | 9483 | 98.04 |  | 596986 | 98.27 | 8536 | 97.99 |  | 556709 | 98.18 | 4093 | 97.62 |  |
| **SVN** |  |  |  |  |  |  |  |  |  |  |  |  |  |  |  |  |  |  |  |  |  |  |  |  |  |
| Yes | 588020 | 91.29 | 3491 | 90.49 | 0.0843 | 572273 | 90.98 | 10378 | 90.8 | 0.5982 | 563323 | 91.31 | 8885 | 91.85 | 0.0624 | 550675 | 90.64 | 7938 | 91.13 | 0.1296 | 510443 | 90.02 | 3780 | 90.15 | 0.7996 |
| No | 56127 | 8.71 | 367 | 9.51 |  | 56717 | 9.02 | 1047 | 9.16 |  | 53607 | 8.69 | 788 | 8.15 |  | 56838 | 9.36 | 773 | 8.87 |  | 56588 | 9.98 | 413 | 9.85 |  |
| **LBW** |  |  |  |  |  |  |  |  |  |  |  |  |  |  |  |  |  |  |  |  |  |  |  |  |  |
| Yes | 585318 | 90.87 | 3467 | 89.87 | 0.0336 | 569765 | 90.58 | 10333 | 90.4 | 0.6176 | 560947 | 90.93 | 8840 | 91.39 | 0.1199 | 548339 | 90.26 | 7900 | 90.69 | 0.1845 | 508028 | 89.59 | 3757 | 89.6 | 1.0000 |
| No | 58829 | 9.13 | 391 | 10.13 |  | 59225 | 9.42 | 1092 | 9.56 |  | 55983 | 9.07 | 833 | 8.61 |  | 59174 | 9.74 | 811 | 9.31 |  | 59003 | 10.41 | 436 | 10.4 |  |
| **PB** |  |  |  |  |  |  |  |  |  |  |  |  |  |  |  |  |  |  |  |  |  |  |  |  |  |
| Yes | 582838 | 90.48 | 3481 | 90.23 | 0.6111 | 567985 | 90.3 | 10316 | 90.3 | 0.9902 | 557587 | 90.38 | 8754 | 90.5 | 0.708 | 543296 | 89.43 | 7809 | 89.65 | 0.5269 | 504798 | 89.02 | 3746 | 89.34 | 0.5322 |
| No | 61309 | 9.52 | 377 | 9.77 |  | 61005 | 9.7 | 1109 | 9.71 |  | 59343 | 9.62 | 919 | 9.5 |  | 64217 | 10.57 | 902 | 10.35 |  | 62233 | 10.98 | 447 | 10.66 |  |

**Appendix 2A. Interaction terms between maternal origin and key sociodemographic and reproductive variables (SVN)**

| **Variable** | **SVN** | | | | | | | | | | | | | |  |  |  |  |  |
| --- | --- | --- | --- | --- | --- | --- | --- | --- | --- | --- | --- | --- | --- | --- | --- | --- | --- | --- | --- |
|  | **OR** | **CI_lower** | **CI_upper** | **p-value** |  | **OR** | **CI_lower** | **CI_upper** | **p-value** |  | **OR** | **CI_lower** | **CI_upper** | **p-value** |  | **OR** | **CI_lower** | **CI_upper** | **p-value** |
| **Maternal origin** |  |  |  |  |  |  |  |  |  |  |  |  |  |  |  |  |  |  |  |
| Venezuela | 1.005 | 0.838 | 1.204 | 0.9595 |  | 1.098 | 1.012 | 1.191 | 0.024 |  | 1.158 | 1.11 | 1.209 | <0.0001 |  | 1.106 | 1.000 | 1.225 | 0.051 |
| **Maternal age groups (years)** |  |  |  |  |  |  |  |  |  |  |  |  |  |  |  |  |  |  |  |
| 10-19 | 0.901 | 0.886 | 0.917 | <0.0001 |  | 0.901 | 0.886 | 0.916 | <0.0001 |  | 0.901 | 0.886 | 0.916 | <0.0001 |  | 0.901 | 0.886 | 0.917 | <0.0001 |
| 20-34 | 0.792 | 0.782 | 0.803 | <0.0001 |  | 0.792 | 0.782 | 0.803 | <0.0001 |  | 0.792 | 0.782 | 0.803 | <0.0001 |  | 0.792 | 0.782 | 0.803 | <0.0001 |
| **Maternal education** |  |  |  |  |  |  |  |  |  |  |  |  |  |  |  |  |  |  |  |
| Secondary | 0.912 | 0.901 | 0.924 | <0.0001 |  | 0.911 | 0.9 | 0.923 | <0.0001 |  | 0.912 | 0.901 | 0.924 | <0.0001 |  | 0.912 | 0.901 | 0.924 | <0.0001 |
| Technical and technological | 0.881 | 0.866 | 0.896 | <0.0001 |  | 0.88 | 0.865 | 0.895 | <0.0001 |  | 0.881 | 0.866 | 0.896 | <0.0001 |  | 0.881 | 0.866 | 0.896 | <0.0001 |
| Professional or higher | 0.746 | 0.733 | 0.759 | <0.0001 |  | 0.745 | 0.732 | 0.758 | <0.0001 |  | 0.746 | 0.733 | 0.759 | <0.0001 |  | 0.746 | 0.733 | 0.759 | <0.0001 |
| No information | 0.967 | 0.94 | 0.996 | 0.025 |  | 0.959 | 0.932 | 0.988 | 0.0057 |  | 0.967 | 0.94 | 0.996 | 0.0253 |  | 0.967 | 0.939 | 0.995 | 0.0233 |
| **Marital status** |  |  |  |  |  |  |  |  |  |  |  |  |  |  |  |  |  |  |  |
| Single/divorced/widow | 1.142 | 1.129 | 1.155 | <0.0001 |  | 1.142 | 1.129 | 1.155 | <0.0001 |  | 1.143 | 1.13 | 1.156 | <0.0001 |  | 1.142 | 1.129 | 1.155 | <0.0001 |
| No information | 0.943 | 0.913 | 0.973 | 0.0003 |  | 0.939 | 0.91 | 0.97 | <0.0001 |  | 0.93 | 0.9 | 0.96 | <0.0001 |  | 0.941 | 0.911 | 0.971 | 0.0002 |
| **Region** |  |  |  |  |  |  |  |  |  |  |  |  |  |  |  |  |  |  |  |
| Caribe | 0.548 | 0.541 | 0.554 | <0.0001 |  | 0.548 | 0.541 | 0.554 | <0.0001 |  | 0.548 | 0.541 | 0.554 | <0.0001 |  | 0.548 | 0.541 | 0.554 | <0.0001 |
| Central | 0.532 | 0.526 | 0.539 | <0.0001 |  | 0.532 | 0.526 | 0.539 | <0.0001 |  | 0.532 | 0.526 | 0.539 | <0.0001 |  | 0.532 | 0.526 | 0.539 | <0.0001 |
| Oriental | 0.507 | 0.5 | 0.514 | <0.0001 |  | 0.506 | 0.499 | 0.513 | <0.0001 |  | 0.506 | 0.499 | 0.513 | <0.0001 |  | 0.506 | 0.499 | 0.513 | <0.0001 |
| Pacifica | 0.546 | 0.539 | 0.554 | <0.0001 |  | 0.546 | 0.539 | 0.554 | <0.0001 |  | 0.547 | 0.539 | 0.554 | <0.0001 |  | 0.546 | 0.539 | 0.554 | <0.0001 |
| Orinoquia, Amazonia | 0.306 | 0.297 | 0.315 | <0.0001 |  | 0.316 | 0.307 | 0.325 | <0.0001 |  | 0.316 | 0.307 | 0.325 | <0.0001 |  | 0.315 | 0.306 | 0.325 | <0.0001 |
| **Newborn sex** |  |  |  |  |  |  |  |  |  |  |  |  |  |  |  |  |  |  |  |
| Female | 1.211 | 1.202 | 1.221 | <0.0001 |  | 1.211 | 1.202 | 1.221 | <0.0001 |  | 1.211 | 1.202 | 1.221 | <0.0001 |  | 1.211 | 1.202 | 1.221 | <0.0001 |
| **Number of previous pregnancies** | |  |  |  |  |  |  |  |  |  |  |  |  |  |  |  |  |  |  |
| 1 | 0.856 | 0.834 | 0.878 | <0.0001 |  | 0.857 | 0.835 | 0.879 | <0.0001 |  | 0.857 | 0.835 | 0.879 | <0.0001 |  | 0.857 | 0.835 | 0.879 | <0.0001 |
| 2-4 | 0.97 | 0.952 | 0.989 | 0.002 |  | 0.971 | 0.952 | 0.989 | 0.0024 |  | 0.971 | 0.952 | 0.989 | 0.0024 |  | 0.971 | 0.952 | 0.99 | 0.0025 |
| **Interbirth interval** |  |  |  |  |  |  |  |  |  |  |  |  |  |  |  |  |  |  |  |
| 0-2 years | 1.052 | 1.033 | 1.07 | <0.0001 |  | 1.052 | 1.033 | 1.07 | <0.0001 |  | 1.052 | 1.033 | 1.07 | <0.0001 |  | 1.048 | 1.029 | 1.067 | <0.0001 |
| 2.1 years - 5 years | 0.878 | 0.867 | 0.89 | <0.0001 |  | 0.878 | 0.867 | 0.89 | <0.0001 |  | 0.878 | 0.867 | 0.889 | <0.0001 |  | 0.877 | 0.866 | 0.889 | <0.0001 |
| Primiparous | 1.402 | 1.376 | 1.428 | <0.0001 |  | 1.402 | 1.377 | 1.428 | <0.0001 |  | 1.402 | 1.376 | 1.428 | <0.0001 |  | 1.402 | 1.377 | 1.428 | <0.0001 |
| **Multiple birth** |  |  |  |  |  |  |  |  |  |  |  |  |  |  |  |  |  |  |  |
| No | 0.047 | 0.046 | 0.048 | <0.0001 |  | 0.047 | 0.046 | 0.048 | <0.0001 |  | 0.047 | 0.046 | 0.048 | <0.0001 |  | 0.047 | 0.046 | 0.048 | <0.0001 |
| Survey year |  |  |  |  |  |  |  |  |  |  |  |  |  |  |  |  |  |  |  |
| 2019 | 1.053 | 1.04 | 1.066 | <0.0001 |  | 1.052 | 1.039 | 1.066 | <0.0001 |  | 1.052 | 1.039 | 1.066 | <0.0001 |  | 1.053 | 1.04 | 1.066 | <0.0001 |
| 2020 | 1.014 | 1.001 | 1.027 | 0.0332 |  | 1.015 | 1.002 | 1.028 | 0.0263 |  | 1.015 | 1.002 | 1.028 | 0.0267 |  | 1.014 | 1.001 | 1.028 | 0.0291 |
| 2021 | 1.126 | 1.111 | 1.14 | <0.0001 |  | 1.126 | 1.112 | 1.141 | <0.0001 |  | 1.126 | 1.112 | 1.141 | <0.0001 |  | 1.126 | 1.112 | 1.141 | <0.0001 |
| 2022 | 1.204 | 1.189 | 1.22 | <0.0001 |  | 1.205 | 1.19 | 1.22 | <0.0001 |  | 1.205 | 1.19 | 1.22 | <0.0001 |  | 1.205 | 1.189 | 1.22 | <0.0001 |
| **Interaction: Mother origin × Region** | | | | | | | | | | | | | |  |  |  |  |  |  |
| Venezuela:Caribe | 1.118 | 0.924 | 1.354 | 0.2505 |  |  |  |  |  |  |  |  |  |  |  |  |  |  |  |
| Venezuela:Central | 0.862 | 0.534 | 1.39 | 0.5413 |  |  |  |  |  |  |  |  |  |  |  |  |  |  |  |
| Venezuela:Oriental | 1.103 | 0.911 | 1.337 | 0.3154 |  |  |  |  |  |  |  |  |  |  |  |  |  |  |  |
| Venezuela:Pacifica | 0.81 | 0.369 | 1.78 | 0.5998 |  |  |  |  |  |  |  |  |  |  |  |  |  |  |  |
| Venezuela:Orinoquia, Amazonia | 1.536 | 1.257 | 1.876 | <0.0001 |  |  |  |  |  |  |  |  |  |  |  |  |  |  |  |
| **Interaction: Mother origin × Maternal education** | | | | | | | | |  |  |  |  |  |  |  |  |  |  |  |
| Venezuela:Secondary |  |  |  |  |  | 1.056 | 0.961 | 1.159 | 0.2561 |  |  |  |  |  |  |  |  |  |  |
| Venezuela:Technical and technological | |  |  |  |  | 0.966 | 0.73 | 1.278 | 0.8075 |  |  |  |  |  |  |  |  |  |  |
| Venezuela:Professional or higher |  |  |  |  |  | 0.966 | 0.73 | 1.278 | 0.8075 |  |  |  |  |  |  |  |  |  |  |
| Venezuela:No information |  |  |  |  |  | 1.321 | 1.146 | 1.523 | <0.0001 |  |  |  |  |  |  |  |  |  |  |
| **Interaction: Mother origin × Marital status** | | | | | | | | | | | | | | | | | | | |
| Venezuela:Single/divorced/widowed |  |  |  |  |  |  |  |  |  |  | 0.934 | 0.839 | 1.041 | 0.2197 |  |  |  |  |  |
| Venezuela:No information |  |  |  |  |  |  |  |  |  |  | 1.303 | 1.149 | 1.478 | <0.0001 |  |  |  |  |  |
| **Interaction: Mother origin × Interbirth interval** | | | | | | | | | | | | | | | | | | | |
| Venezuela:0-2 years |  |  |  |  |  |  |  |  |  |  |  |  |  |  |  | 1.196 | 1.041 | 1.374 | 0.0116 |
| Venezuela:2.1 years - 5 years |  |  |  |  |  |  |  |  |  |  |  |  |  |  |  | 1.109 | 0.98 | 1.254 | 0.1006 |
| Venezuela:Primiparous |  |  |  |  |  |  |  |  |  |  |  |  |  |  |  | 1.013 | 0.902 | 1.137 | 0.8328 |

**Appendix 2A. Interaction terms between maternal origin and key sociodemographic and reproductive variables (LBW)**

| **Variable** | **LBW** | | | | | | | | | | | | | |  |  |  |  |  |
| --- | --- | --- | --- | --- | --- | --- | --- | --- | --- | --- | --- | --- | --- | --- | --- | --- | --- | --- | --- |
|  | **OR** | **CI_lower** | **CI_upper** | **p-value** |  | **OR** | **CI_lower** | **CI_upper** | **p-value** |  | **OR** | **CI_lower** | **CI_upper** | **p-value** |  | **OR** | **CI_lower** | **CI_upper** | **p-value** |
| **Maternal origin** |  |  |  |  |  |  |  |  |  |  |  |  |  |  |  |  |  |  |  |
| Venezuela | 1.014 | 0.849 | 1.212 | 0.874 |  | 1.122 | 1.037 | 1.215 | 0.0044 |  | 1.17 | 1.122 | 1.221 | <0.0001 |  | 1.129 | 1.022 | 1.247 | 0.0165 |
| **Maternal age groups (years)** | |  |  |  |  |  |  |  |  |  |  |  |  |  |  |  |  |  |  |
| 10-19 | 0.883 | 0.868 | 0.898 | <0.0001 |  | 0.882 | 0.868 | 0.897 | <0.0001 |  | 0.883 | 0.868 | 0.897 | <0.0001 |  | 0.883 | 0.868 | 0.898 | <0.0001 |
| 20-34 | 0.784 | 0.774 | 0.794 | <0.0001 |  | 0.784 | 0.774 | 0.794 | <0.0001 |  | 0.784 | 0.774 | 0.794 | <0.0001 |  | 0.784 | 0.774 | 0.794 | <0.0001 |
| **Maternal education** |  |  |  |  |  |  |  |  |  |  |  |  |  |  |  |  |  |  |  |
| Secondary | 0.918 | 0.907 | 0.93 | <0.0001 |  | 0.917 | 0.906 | 0.929 | <0.0001 |  | 0.918 | 0.907 | 0.93 | <0.0001 |  | 0.918 | 0.907 | 0.93 | <0.0001 |
| Technical and technological | 0.885 | 0.871 | 0.9 | <0.0001 |  | 0.885 | 0.87 | 0.9 | <0.0001 |  | 0.885 | 0.871 | 0.9 | <0.0001 |  | 0.886 | 0.871 | 0.9 | <0.0001 |
| Professional or higher | 0.746 | 0.733 | 0.759 | <0.0001 |  | 0.745 | 0.732 | 0.758 | <0.0001 |  | 0.746 | 0.733 | 0.759 | <0.0001 |  | 0.746 | 0.733 | 0.759 | <0.0001 |
| No information | 0.983 | 0.955 | 1.011 | 0.2314 |  | 0.975 | 0.948 | 1.004 | 0.0879 |  | 0.983 | 0.955 | 1.011 | 0.234 |  | 0.982 | 0.955 | 1.011 | 0.2209 |
| **Marital status** |  |  |  |  |  |  |  |  |  |  |  |  |  |  |  |  |  |  |  |
| Single/divorced/widow | 1.149 | 1.136 | 1.162 | <0.0001 |  | 1.149 | 1.136 | 1.162 | <0.0001 |  | 1.15 | 1.137 | 1.163 | <0.0001 |  | 1.149 | 1.136 | 1.162 | <0.0001 |
| No information | 0.951 | 0.922 | 0.981 | 0.0016 |  | 0.948 | 0.919 | 0.978 | 0.0008 |  | 0.938 | 0.908 | 0.968 | 0.0001 |  | 0.949 | 0.92 | 0.979 | 0.001 |
| **Region** |  |  |  |  |  |  |  |  |  |  |  |  |  |  |  |  |  |  |  |
| Caribe | 0.55 | 0.543 | 0.556 | <0.0001 |  | 0.55 | 0.543 | 0.556 | <0.0001 |  | 0.55 | 0.543 | 0.556 | <0.0001 |  | 0.549 | 0.543 | 0.556 | <0.0001 |
| Central | 0.532 | 0.525 | 0.538 | <0.0001 |  | 0.532 | 0.525 | 0.538 | <0.0001 |  | 0.532 | 0.525 | 0.538 | <0.0001 |  | 0.532 | 0.525 | 0.539 | <0.0001 |
| Oriental | 0.504 | 0.497 | 0.511 | <0.0001 |  | 0.503 | 0.496 | 0.51 | <0.0001 |  | 0.503 | 0.496 | 0.51 | <0.0001 |  | 0.504 | 0.497 | 0.51 | <0.0001 |
| Pacifica | 0.554 | 0.546 | 0.562 | <0.0001 |  | 0.554 | 0.546 | 0.562 | <0.0001 |  | 0.554 | 0.546 | 0.562 | <0.0001 |  | 0.554 | 0.546 | 0.562 | <0.0001 |
| Orinoquia, Amazonia | 0.301 | 0.292 | 0.31 | <0.0001 |  | 0.311 | 0.302 | 0.32 | <0.0001 |  | 0.311 | 0.302 | 0.32 | <0.0001 |  | 0.311 | 0.302 | 0.32 | <0.0001 |
| **Newborn sex** |  |  |  |  |  |  |  |  |  |  |  |  |  |  |  |  |  |  |  |
| Female | 1.206 | 1.197 | 1.216 | <0.0001 |  | 1.206 | 1.197 | 1.216 | <0.0001 |  | 1.206 | 1.197 | 1.216 | <0.0001 |  | 1.206 | 1.197 | 1.216 | <0.0001 |
| **Number of previous pregnancies** | |  |  |  |  |  |  |  |  |  |  |  |  |  |  |  |  |  |  |
| 1 | 0.832 | 0.812 | 0.854 | <0.0001 |  | 0.833 | 0.812 | 0.854 | <0.0001 |  | 0.833 | 0.812 | 0.854 | <0.0001 |  | 0.833 | 0.812 | 0.854 | <0.0001 |
| 2-4 | 0.973 | 0.954 | 0.991 | 0.0041 |  | 0.973 | 0.955 | 0.992 | 0.0048 |  | 0.973 | 0.955 | 0.992 | 0.0048 |  | 0.973 | 0.955 | 0.992 | 0.0051 |
| **Interbirth interval** |  |  |  |  |  |  |  |  |  |  |  |  |  |  |  |  |  |  |  |
| 0-2 years | 1.061 | 1.043 | 1.08 | <0.0001 |  | 1.061 | 1.043 | 1.08 | <0.0001 |  | 1.061 | 1.043 | 1.08 | <0.0001 |  | 1.057 | 1.039 | 1.076 | <0.0001 |
| 2.1 years - 5 years | 0.875 | 0.864 | 0.886 | <0.0001 |  | 0.875 | 0.864 | 0.886 | <0.0001 |  | 0.875 | 0.864 | 0.886 | <0.0001 |  | 0.874 | 0.863 | 0.885 | <0.0001 |
| Primiparous | 1.45 | 1.424 | 1.477 | <0.0001 |  | 1.451 | 1.425 | 1.477 | <0.0001 |  | 1.45 | 1.425 | 1.477 | <0.0001 |  | 1.451 | 1.425 | 1.477 | <0.0001 |
| **Multiple birth** |  |  |  |  |  |  |  |  |  |  |  |  |  |  |  |  |  |  |  |
| No | 0.042 | 0.042 | 0.043 | <0.0001 |  | 0.042 | 0.042 | 0.043 | <0.0001 |  | 0.042 | 0.042 | 0.043 | <0.0001 |  | 0.042 | 0.042 | 0.043 | <0.0001 |
| Survey year |  |  |  |  |  |  |  |  |  |  |  |  |  |  |  |  |  |  |  |
| 2019 | 1.048 | 1.035 | 1.062 | <0.0001 |  | 1.048 | 1.035 | 1.061 | <0.0001 |  | 1.048 | 1.035 | 1.061 | <0.0001 |  | 1.048 | 1.035 | 1.062 | <0.0001 |
| 2020 | 1.01 | 0.997 | 1.022 | 0.1401 |  | 1.01 | 0.997 | 1.023 | 0.1164 |  | 1.01 | 0.997 | 1.023 | 0.1172 |  | 1.01 | 0.997 | 1.023 | 0.1256 |
| 2021 | 1.117 | 1.104 | 1.131 | <0.0001 |  | 1.118 | 1.104 | 1.132 | <0.0001 |  | 1.118 | 1.104 | 1.132 | <0.0001 |  | 1.118 | 1.104 | 1.132 | <0.0001 |
| 2022 | 1.198 | 1.184 | 1.214 | <0.0001 |  | 1.199 | 1.184 | 1.214 | <0.0001 |  | 1.199 | 1.184 | 1.214 | <0.0001 |  | 1.199 | 1.184 | 1.214 | <0.0001 |
| **Interaction: Mother origin × Region** | | | | | | | | | | | | | |  |  |  |  |  |  |
| Venezuela:Caribe | 1.119 | 0.928 | 1.349 | 0.2393 |  |  |  |  |  |  |  |  |  |  |  |  |  |  |  |
| Venezuela:Central | 0.899 | 0.568 | 1.424 | 0.6502 |  |  |  |  |  |  |  |  |  |  |  |  |  |  |  |
| Venezuela:Oriental | 1.108 | 0.918 | 1.338 | 0.2854 |  |  |  |  |  |  |  |  |  |  |  |  |  |  |  |
| Venezuela:Pacifica | 0.875 | 0.417 | 1.837 | 0.7242 |  |  |  |  |  |  |  |  |  |  |  |  |  |  |  |
| Venezuela:Orinoquia, Amazonia | 1.558 | 1.281 | 1.896 | <0.0001 |  |  |  |  |  |  |  |  |  |  |  |  |  |  |  |
| **Interaction: Mother origin × Maternal education** | | | | | | | | |  |  |  |  |  |  |  |  |  |  |  |
| Venezuela:Secondary |  |  |  |  |  | 1.045 | 0.954 | 1.146 | 0.344 |  |  |  |  |  |  |  |  |  |  |
| Venezuela:Technical and technological | |  |  |  |  | 0.95 | 0.721 | 1.25 | 0.7129 |  |  |  |  |  |  |  |  |  |  |
| Venezuela:Professional or higher | |  |  |  |  | 1.274 | 1.041 | 1.559 | 0.0188 |  |  |  |  |  |  |  |  |  |  |
| Venezuela:No information |  |  |  |  |  | 1.297 | 1.128 | 1.491 | 0.0003 |  |  |  |  |  |  |  |  |  |  |
| **Interaction: Mother origin × Marital status** | | | | | | | | | | | | | | | | | | | |
| Venezuela:Single/divorced/widowed | |  |  |  |  |  |  |  |  |  | 0.958 | 0.862 | 1.065 | 0.4273 |  |  |  |  |  |
| Venezuela:No information |  |  |  |  |  |  |  |  |  |  | 1.308 | 1.156 | 1.48 | <0.0001 |  |  |  |  |  |
| **Interaction: Mother origin × Interbirth interval** | | | | | | | | | | | | | | | | | | | |
| Venezuela:0-2 years |  |  |  |  |  |  |  |  |  |  |  |  |  |  |  | 1.201 | 1.048 | 1.375 | 0.0082 |
| Venezuela:2.1 years - 5 years | |  |  |  |  |  |  |  |  |  |  |  |  |  |  | 1.1 | 0.975 | 1.241 | 0.1208 |
| Venezuela: Primiparous |  |  |  |  |  |  |  |  |  |  |  |  |  |  |  | 1.002 | 0.894 | 1.122 | 0.9794 |

**Appendix 2A. Interaction terms between maternal origin and key sociodemographic and reproductive variables (PB)**

| **Variable** | **LBW** | | | | | | | | | | | | | |  |  |  |  |  |
| --- | --- | --- | --- | --- | --- | --- | --- | --- | --- | --- | --- | --- | --- | --- | --- | --- | --- | --- | --- |
|  | **OR** | **CI_lower** | **CI_upper** | **p-value** |  | **OR** | **CI_lower** | **CI_upper** | **p-value** |  | **OR** | **CI_lower** | **CI_upper** | **p-value** |  | **OR** | **CI_lower** | **CI_upper** | **p-value** |
| **Maternal origin** |  |  |  |  |  |  |  |  |  |  |  |  |  |  |  |  |  |  |  |
| Venezuela | 1.006 | 0.828 | 1.221 | 0.9527 |  | 0.956 | 0.884 | 1.034 | 0.2623 |  | 1.028 | 0.986 | 1.071 | 0.1908 |  | 0.998 | 0.908 | 1.098 | 0.9704 |
| **Maternal age groups (years)** |  |  |  |  |  |  |  |  |  |  |  |  |  |  |  |  |  |  |  |
| 10-19 | 0.995 | 0.979 | 1.011 | 0.539 |  | 0.995 | 0.979 | 1.011 | 0.5462 |  | 0.995 | 0.979 | 1.012 | 0.558 |  | 0.995 | 0.979 | 1.012 | 0.5652 |
| 20-34 | 0.832 | 0.822 | 0.843 | <0.0001 |  | 0.832 | 0.822 | 0.842 | <0.0001 |  | 0.832 | 0.822 | 0.843 | <0.0001 |  | 0.832 | 0.822 | 0.843 | <0.0001 |
| **Maternal education** |  |  |  |  |  |  |  |  |  |  |  |  |  |  |  |  |  |  |  |
| Secondary | 0.974 | 0.963 | 0.986 | <0.0001 |  | 0.973 | 0.961 | 0.985 | <0.0001 |  | 0.975 | 0.963 | 0.986 | <0.0001 |  | 0.975 | 0.963 | 0.986 | <0.0001 |
| Technical and technological | 1.012 | 0.996 | 1.028 | 0.1386 |  | 1.011 | 0.995 | 1.027 | 0.1861 |  | 1.012 | 0.996 | 1.028 | 0.1279 |  | 1.012 | 0.997 | 1.029 | 0.1269 |
| Professional or higher | 0.931 | 0.916 | 0.947 | <0.0001 |  | 0.929 | 0.914 | 0.945 | <0.0001 |  | 0.931 | 0.916 | 0.947 | <0.0001 |  | 0.932 | 0.916 | 0.947 | <0.0001 |
| No information | 0.935 | 0.909 | 0.961 | <0.0001 |  | 0.928 | 0.902 | 0.954 | <0.0001 |  | 0.936 | 0.91 | 0.962 | <0.0001 |  | 0.935 | 0.91 | 0.962 | <0.0001 |
| **Marital status** |  |  |  |  |  |  |  |  |  |  |  |  |  |  |  |  |  |  |  |
| Single/divorced/widow | 1.131 | 1.118 | 1.144 | <0.0001 |  | 1.131 | 1.119 | 1.144 | <0.0001 |  | 1.13 | 1.117 | 1.143 | <0.0001 |  | 1.131 | 1.119 | 1.144 | <0.0001 |
| No information | 1.014 | 0.984 | 1.044 | 0.3759 |  | 1.011 | 0.981 | 1.041 | 0.4915 |  | 1.006 | 0.976 | 1.037 | 0.6986 |  | 1.012 | 0.982 | 1.043 | 0.4267 |
| **Region** |  |  |  |  |  |  |  |  |  |  |  |  |  |  |  |  |  |  |  |
| Caribe | 0.86 | 0.85 | 0.87 | <0.0001 |  | 0.859 | 0.849 | 0.869 | <0.0001 |  | 0.859 | 0.849 | 0.869 | <0.0001 |  | 0.859 | 0.849 | 0.869 | <0.0001 |
| Central | 0.719 | 0.71 | 0.728 | <0.0001 |  | 0.719 | 0.71 | 0.728 | <0.0001 |  | 0.719 | 0.71 | 0.728 | <0.0001 |  | 0.719 | 0.71 | 0.728 | <0.0001 |
| Oriental | 0.727 | 0.717 | 0.737 | <0.0001 |  | 0.727 | 0.717 | 0.737 | <0.0001 |  | 0.727 | 0.717 | 0.737 | <0.0001 |  | 0.727 | 0.717 | 0.737 | <0.0001 |
| Pacifica | 0.794 | 0.782 | 0.805 | <0.0001 |  | 0.794 | 0.783 | 0.805 | <0.0001 |  | 0.794 | 0.783 | 0.805 | <0.0001 |  | 0.794 | 0.783 | 0.805 | <0.0001 |
| Orinoquia, Amazonia | 0.509 | 0.495 | 0.523 | <0.0001 |  | 0.525 | 0.511 | 0.538 | <0.0001 |  | 0.524 | 0.51 | 0.538 | <0.0001 |  | 0.524 | 0.51 | 0.538 | <0.0001 |
| **Newborn sex** |  |  |  |  |  |  |  |  |  |  |  |  |  |  |  |  |  |  |  |
| Female | 0.928 | 0.921 | 0.936 | <0.0001 |  | 0.928 | 0.921 | 0.936 | <0.0001 |  | 0.928 | 0.921 | 0.936 | <0.0001 |  | 0.928 | 0.921 | 0.936 | <0.0001 |
| **Number of previous pregnancies** | |  |  |  |  |  |  |  |  |  |  |  |  |  |  |  |  |  |  |
| 01-Jan | 0.69 | 0.673 | 0.706 | <0.0001 |  | 0.69 | 0.674 | 0.707 | <0.0001 |  | 0.69 | 0.673 | 0.707 | <0.0001 |  | 0.69 | 0.673 | 0.707 | <0.0001 |
| 02-Apr | 0.902 | 0.886 | 0.918 | <0.0001 |  | 0.902 | 0.887 | 0.918 | <0.0001 |  | 0.902 | 0.887 | 0.918 | <0.0001 |  | 0.902 | 0.887 | 0.918 | <0.0001 |
| **Interbirth interval** |  |  |  |  |  |  |  |  |  |  |  |  |  |  |  |  |  |  |  |
| 0-2 years | 1.2 | 1.181 | 1.219 | <0.0001 |  | 1.2 | 1.181 | 1.219 | <0.0001 |  | 1.2 | 1.181 | 1.219 | <0.0001 |  | 1.195 | 1.176 | 1.214 | <0.0001 |
| 2.1 years - 5 years | 0.931 | 0.92 | 0.942 | <0.0001 |  | 0.931 | 0.92 | 0.942 | <0.0001 |  | 0.931 | 0.92 | 0.942 | <0.0001 |  | 0.931 | 0.92 | 0.942 | <0.0001 |
| Primiparous | 1.287 | 1.264 | 1.309 | <0.0001 |  | 1.287 | 1.265 | 1.31 | <0.0001 |  | 1.287 | 1.264 | 1.31 | <0.0001 |  | 1.287 | 1.264 | 1.309 | <0.0001 |
| **Multiple birth** |  |  |  |  |  |  |  |  |  |  |  |  |  |  |  |  |  |  |  |
| No | 0.049 | 0.048 | 0.05 | <0.0001 |  | 0.049 | 0.048 | 0.05 | <0.0001 |  | 0.049 | 0.048 | 0.05 | <0.0001 |  | 0.049 | 0.048 | 0.05 | <0.0001 |
| Survey year |  |  |  |  |  |  |  |  |  |  |  |  |  |  |  |  |  |  |  |
| 2019 | 1.027 | 1.015 | 1.04 | <0.0001 |  | 1.027 | 1.015 | 1.039 | <0.0001 |  | 1.027 | 1.015 | 1.04 | <0.0001 |  | 1.027 | 1.015 | 1.04 | <0.0001 |
| 2020 | 1.02 | 1.008 | 1.033 | 0.0014 |  | 1.021 | 1.008 | 1.033 | 0.001 |  | 1.021 | 1.008 | 1.033 | 0.0011 |  | 1.02 | 1.008 | 1.033 | 0.0012 |
| 2021 | 1.145 | 1.131 | 1.158 | <0.0001 |  | 1.145 | 1.132 | 1.159 | <0.0001 |  | 1.145 | 1.132 | 1.159 | <0.0001 |  | 1.145 | 1.131 | 1.159 | <0.0001 |
| 2022 | 1.191 | 1.177 | 1.206 | <0.0001 |  | 1.192 | 1.178 | 1.207 | <0.0001 |  | 1.192 | 1.178 | 1.207 | <0.0001 |  | 1.192 | 1.177 | 1.206 | <0.0001 |
| **Interaction: Mother origin × Region** | | | | | | | | | | | | | |  |  |  |  |  |  |
| Venezuela:Caribe | 0.927 | 0.757 | 1.136 | 0.4659 |  |  |  |  |  |  |  |  |  |  |  |  |  |  |  |
| Venezuela:Central | 0.586 | 0.342 | 1.004 | 0.0519 |  |  |  |  |  |  |  |  |  |  |  |  |  |  |  |
| Venezuela:Oriental | 1.044 | 0.852 | 1.28 | 0.6761 |  |  |  |  |  |  |  |  |  |  |  |  |  |  |  |
| Venezuela:Pacifica | 1.212 | 0.646 | 2.276 | 0.5493 |  |  |  |  |  |  |  |  |  |  |  |  |  |  |  |
| Venezuela:Orinoquia, Amazonia | 1.401 | 1.137 | 1.726 | 0.0015 |  |  |  |  |  |  |  |  |  |  |  |  |  |  |  |
| **Interaction: Mother origin × Maternal education** | | | | | | | | |  |  |  |  |  |  |  |  |  |  |  |
| Venezuela:Secondary |  |  |  |  |  | 1.101 | 1.007 | 1.205 | 0.0352 |  |  |  |  |  |  |  |  |  |  |
| Venezuela:Technical and technological | |  |  |  |  | 1.005 | 0.772 | 1.307 | 0.9718 |  |  |  |  |  |  |  |  |  |  |
| Venezuela:Professional or higher |  |  |  |  |  | 1.275 | 1.052 | 1.546 | 0.0134 |  |  |  |  |  |  |  |  |  |  |
| Venezuela:No information |  |  |  |  |  | 1.381 | 1.203 | 1.585 | 0 |  |  |  |  |  |  |  |  |  |  |
| **Interaction: Mother origin × Marital status** | | | | | | | | | | | | | | | | | | | |
| Venezuela:Single/divorced/widowed |  |  |  |  |  |  |  |  |  |  | 1.103 | 0.998 | 1.219 | 0.0536 |  |  |  |  |  |
| Venezuela:No information |  |  |  |  |  |  |  |  |  |  | 1.172 | 1.034 | 1.328 | 0.0131 |  |  |  |  |  |
| **Interaction: Mother origin × Interbirth interval** | | | | | | | | | | | | | | | | | | | |
| Venezuela:0-2 years |  |  |  |  |  |  |  |  |  |  |  |  |  |  |  | 1.231 | 1.085 | 1.397 | 0.0013 |
| Venezuela:2.1 years - 5 years |  |  |  |  |  |  |  |  |  |  |  |  |  |  |  | 1.029 | 0.917 | 1.155 | 0.6252 |
| Venezuela:Primiparous |  |  |  |  |  |  |  |  |  |  |  |  |  |  |  | 1.034 | 0.926 | 1.155 | 0.5513 |
